# Trends in unprotected intercourse among heterosexual men before and after brothel ban in Siem Reap, Cambodia: a serial cross-sectional study (2003–2012)

**DOI:** 10.1186/s12889-018-5321-0

**Published:** 2018-03-27

**Authors:** Mee Lian Wong, Alvin Kuo Jing Teo, Bee Choo Tai, Alwyn Mao Tong Ng, Raymond Boon Tar Lim, Dede Kam Tyng Tham, Nashwinder Kaur, Rayner Kay Jin Tan, Sarath Kros, Savun Touch, Maryan Chhit, Ian Lubek

**Affiliations:** 1 Saw Swee Hock School of Public Health, National University of Singapore, Tahir Foundation Building, 12 Science Drive 2 #10-01, Singapore, 117549 Singapore; 2 Provincial Health Department, Siem Reap Province, Cambodia; 3 Provincial AIDS Office, Siem Reap Province, Cambodia; 4 Khmer Soviet Friendship Hospital, Phnom Penh Province, Cambodia; 5 Department of Psychology, University of Guelph, Guelph, Canada

**Keywords:** Brothel ban, Heterosexual men buying sex, Unprotected vaginal intercourse, Serial cross-sectional study (2003–2012)

## Abstract

**Background:**

Following Cambodia’s implementation of the 100% condom use program with enforcement of condom use and STI treatment services for sex workers in 2001, sexually transmitted infection and HIV declined markedly. In 2008, Cambodia implemented a law to ban brothel-based sex work. We reported trends in unprotected vaginal intercourse with sex workers among heterosexual men buying sex before (2003**–**2008) and after (2009**–**2012) the brothel ban in Cambodia. We also determined the association of brothel ban with these men’s reports of unprotected intercourse with sex workers.

**Methods:**

In this serial cross-sectional study, we collected yearly behavioural data on random cross-sectional samples of heterosexual men buying sex who attended the only government health centre in Siem Reap for voluntary confidential counselling and testing (VCCT) between 2003 and 2012. We used multivariable Poisson regression analysis on the 10-year data of 976 men to obtain the adjusted prevalence ratio (aPR) of unprotected intercourse in the last 6 months by brothel closure.

**Results:**

Men buying sex from non-brothel-based sex workers increased almost 3-fold from 17% in 2007**–**2008 before brothel closure to 55% in 2011**–**2012 after brothel closure (*p* < 0.001). Unprotected intercourse with sex workers in the last week increased significantly from 37% (2003**–**2004) before brothel closure to 65% (2011**–**2012) after brothel closure. This increase corresponded closely with the increase in self-reported unprotected intercourse from 35% to 61% by the sex workers (*n* = 1805) attending the same clinic for VCCT. Brothel closure was associated with an increased risk (aPR: 1.65; 95% CI: 1.40**–**1.94) of unprotected intercourse with sex workers. HIV prevalence in the heterosexual men declined significantly from 26% in 2003**–**2004 to 4.8% in 2007**–**2008 and 0 case in 2009**–**2010 before increasing to 5.6% in 2011**–**2012.

**Conclusion:**

Our findings suggest that the brothel ban had led to an increase in unprotected intercourse with all sex workers for men buying sex. This effect could be attributed to reduced condom access, a consequence of the lack of feasibility to implement the 100% condom use program following the brothel ban. The ban on brothels in Cambodia should be reviewed.

## Background

Asia is the second most affected region by human immunodeficiency virus (HIV) [1] and South, and Southeast Asia report the highest number of sexually transmitted infections (STIs) worldwide [2]. Male patronage of female sex workers has been reported as the main source of transmission of HIV/STIs in Asia [3]. To control the spread of HIV/STIs, measures ranging from legalisation of sex work [4], medical surveillance [5] to criminalisation [6–8] of sex work have been implemented in several Asian countries. In Goa, India, for example, brothels were demolished by the local state government. However, it did not lead to a decline in commercial sex [6]. Instead, it led to dispersed and clandestine sex work with some working from the slums under the control of pimps, and others working from home or on the streets. More importantly, demolition of the red light areas, forced closures of the brothels and bars as well as criminalisation of sex workers drove sex work underground and led to reduced and difficult access to HIV/STI prevention, testing and treatment services. Consequently, it resulted in negative public health outcomes such as low condom use rates and high rates of STIs [9].

In 2001, Cambodia implemented the 100% condom use program with the promotion and enforcement of condom use predominantly targeted at brothels. Other activities under the program included provision of monthly free screening and STI treatment services at designated health centres to all brothel-based sex workers [4, 10], manager training in supporting sex workers on condom use, and venue monitoring with fines and penalties for non-compliant brothels. For example, a brothel would be temporarily closed if sex workers in that specific brothel tested positive for STIs for three consecutive times during their monthly STI check-ups [10]. National surveillance surveys [10–14] showed a marked decline in STIs [10, 11] and HIV prevalence, with a significant decline in commercial sex transactions and an increase in condom use. Access to HIV voluntary confidential counselling and testing (VCCT) and antiretroviral therapy had also increased. The success attributed to the 100% condom use program resulted in the presentation of the Millennium Development Goals Award to Cambodia in 2010 [15]. However, HIV and STIs remain concentrated in female sex workers [10, 16–18], with heterosexual sex as the main route of transmission [10, 16, 17, 19].

In February 2008, Cambodia implemented a Law on Suppression of Human Trafficking and Sexual Exploitation with the intention to protect the rights and dignity of human beings. However, the new law also resulted in the ban on brothel-based sex work [20, 21] and criminalisation of sex work leading to sex workers facing more harassment and arrests [21]. Studies were conducted by researchers [21, 22] and non-governmental organisations [23, 24] to evaluate the impact of the law. Most of the studies found negative outcomes such as displacement of transactional sex to hidden settings resulting in difficult access to HIV/STI prevention and treatment services. Monitoring by the government also found that sex workers were moving underground to engage in indirect sex work, and self-reported condom use had declined among them [20]. Page et al.’s study [22] compared cohorts of women who engaged in transactional sex before and after the implementation of the brothel ban. The cohort recruited after the ban showed lower HIV prevalence compared to the cohort recruited before the ban. The cohort recruited after the ban however differed significantly from the cohort recruited before the ban in having fewer freelance sex workers (19% vs 30%) but more entertainment workers who worked under a manager (82% vs 46%). Consistent condom use with last paying partner did not differ in the two cohorts. The authors attributed the lower HIV risk in the ‘post-brothel ban’ cohort to fewer paying partners, shorter work duration and the protective effects of entertainment-based work. The same study also found that more brothel-based sex workers were working in entertainment-based venues (68% versus 31%) and reporting more alcohol use which might increase their risk of unprotected sex and STIs.

To the best of our knowledge, published studies [6–8, 21, 23, 24] on the criminalisation of sex work and the ban of brothels and were conducted among female sex workers and its impact was examined at the individual level. Most of the studies were also qualitative [6–8, 21, 24], perhaps due to the difficulty in accessing illegal sex workers for representative surveys. Notably, these qualitative studies provided important insights on how brothel closure had led to unintended negative public health outcomes such as unsafe work environments, sexual risk behaviours and increased HIV/STI risk among the sex workers. These findings were consistent with a recent study on the ecological analysis of criminalisation of sex work in European countries which found its association with higher HIV prevalence [25].

Presently, it remains unclear how the ban on brothel-based sex work has impacted the sexual risk behaviours and HIV risk of male clients of female sex workers. Sex workers constitute only one side of the equation in commercial sexual transactions. To have a better understanding on how brothel closure affects sexual risk behaviours in commercial sexual transactions, it is critical to study the behaviours of male clients of female sex workers. National behavioural sentinel surveillance (BSS) surveys have been conducted regularly in Cambodia until 2010 [14] to collect data on men who were more likely to engage in transactional sex (police, military and motor-taxi drivers). However, these men were excluded from the BSS in 2013 [26], owing to the shift in surveillance to most-at-risk populations for STI/HIV. Behavioural data on men who engaged in transactional sex have also been gathered through the Demographic Health Survey. However, the survey focused on men aged between 25 and 29 years and condom use at last intercourse [27, 28]. Given that men older than 29 years also buy sex, and condom use at last intercourse may not reflect usual sexual risk behaviour, a considerable knowledge gap still exists on sexual risk behaviours of these men particularly after the implementation of the law to ban brothels. It is important to study this priority group of men because they may act as a “bridge” [29] for transmitting STIs/HIV from female sex workers to their wives, girlfriends and other sex workers. The aim of this study was to report temporal trends in unprotected intercourse with sex workers among heterosexual men who bought sex before and after closure of the brothels in 2008. We have been collecting behavioural data on heterosexual men buying sex from 2003 to 2012 as part of our continual monitoring of the educational activities which have been designed and implemented for female entertainment workers by our team. The closure of the brothels in Cambodia in 2008 provides a natural experiment to assess its impact on the behaviours of these men and the sex workers. We hypothesised that brothel closure is associated with an increase in unprotected intercourse in transactional sex among heterosexual men who buy sex.

## Methods

We conducted a serial cross-sectional study with yearly data (2003 to 2012) collected on random samples of heterosexual men and female sex workers attending Mondol Moi Health Centre, the only government health centre for VCCT in Siem Reap. Eligibility criteria for men were those aged 18 years and older who reported having engaged in transactional sex (defined as sex in exchange for money, goods or services) with a female sex worker in the last 6 months at the time of study enrolment. Men who reported having sex with men were excluded.

We took a sample of about 100 heterosexual men and 180 sex workers yearly. The sample size was based on financial and feasibility considerations. Sampling was guided by the principles that it would result in the most representative sample possible, and yet would be efficient and feasible to implement, given the financial and manpower constraints encountered. To reduce selection and seasonal bias, eligible individuals were randomly selected from all 12 months of the year. To enhance feasibility in the implementation of the survey under real-world conditions, we divided each year into 2 strata, (i) January to June and (ii) July to December, and randomly selected 2-month blocks from each stratum every year. Next, we randomly selected 2 weeks per month from each of these blocks so that all weekdays were covered. As an average of 3 heterosexual men and 6 sex workers attended the clinic for VCCT daily, we interviewed all eligible men and sex workers who came to the clinic on the selected days. About 100 men and 180 sex workers were interviewed yearly, constituting about 10% of the men and 40% of the sex workers (brothel and non-brothel) attending the clinic.

### Measures

The primary outcome was self-reported unprotected vaginal intercourse with sex workers in the last 6 months by the heterosexual men. This was defined as not using a condom in the last 6 months, assessed by the question: “Were condoms used in the last 6 months with sex workers?” with three options—yes always, never, sometimes. Those who responded ‘never’ and ‘sometimes’ were categorised as unprotected intercourse. Sex workers were categorised based on their work premise—brothel or non-brothel. Non-brothel based venues included entertainment establishments (beer restaurants, massage parlours, bars, pubs, discotheques) and villas/guesthouses/hotels. The secondary outcomes for heterosexual men included percentage having sex with brothel and/or non-brothel-based sex workers in the last 3 months, mean frequency of unprotected vaginal intercourse with sex workers in the last week, and laboratory-confirmed HIV prevalence. The frequency of unprotected intercourse in the last week was defined as the number of times when a condom was not used. This was computed by subtracting the number of times of intercourse in which a condom was used from the total number of times of intercourse. The period of “last week” was used to increase accuracy of recall.

The primary exposure of interest was status of brothel closure following the criminalisation of soliciting, procurement and management of transactional sex. The Law on Suppression of Human Trafficking and Sexual Exploitation banning brothel based sex work was enforced in February 2008, although qualitative enquiries with key informants found few actual brothel closures in Siem Reap during 2008. As most of the brothels were closed during 2009, we defined the period from 2009 onwards as the post-brothel closure period. Potential confounding variables included alcohol consumption, perceived risk of getting STDs/HIV from sex workers, income and other socio-demographic variables. Participants were defined as ‘drinkers’ if they self-reported alcohol consumption at least once per week. Self-perceived risk of getting STDs/HIV from sex workers was assessed by asking, “What do you think is your chance of getting STDs/HIV from a sex worker/entertainment worker” with 5 response categories—none at all, low, moderate, high and very high. This was categorised as none or low versus moderate to very high. Income was defined as self-reported monthly earnings.

### Data collection

The questionnaire sought participants’ socio-demographic information, sexual partner types, alcohol consumption, condom use, self-perceived risk and knowledge about STDs. Our trained local female counsellor interviewed participants privately in the counselling room, using a Khmer-translated questionnaire, adapted from that used for heterosexual men [30] and sex workers in Singapore [5] and Cambodia [31]. The questionnaire was pre-tested to ensure clarity and cultural appropriateness. To reduce social desirability bias, we assured anonymity and confidentiality, and stressed the importance of responding truthfully because their responses will be used to plan HIV prevention programs to benefit them. The interviewer also explained empathetically to the entertainment workers that she came to know from the national surveys [11, 12] that some of them were engaging in transactional sex; hence, this study aimed to understand their difficulties in using condoms with clients. The VCCT questionnaire-administered study received joint approval from University of Guelph Research Ethics Board (Approval numbers: 06JA002 and 11FE039) and the Provincial Health Department Director. All participants provided oral informed consent for participation. Under the Government’s routine VCCT surveillance program, blood specimens were screened for HIV using enzyme immunoassay (EIA); HIV was confirmed by Determine HIV-1/2 test.

### Statistical analysis

The 10-year study period between 2003 and 2012 was grouped into 5 time periods with 3 time periods before and 2 time periods after brothel closure in 2008. We used Chi-square test for trends to compare, over time, trends in sexual risk behaviours across independent cross-sectional samples.

To assess the independent association of brothel closure with unprotected intercourse in the last 6 months by the men, we conducted multivariable analysis using Poisson regression with robust sandwich variance on the entire 10-year database to obtain the adjusted prevalence ratios (aPR) of unprotected intercourse by brothel closure and potential confounding variables. First, we conducted univariate analyses to determine associations between unprotected vaginal intercourse and brothel closure and other variables. We used the chi-square test, independent-sample t test and Wilcoxon rank-sum test for categorical, continuous, and ordinal variables respectively. All independent variables with *p* < 0.1 in the univariate analyses were further considered for entry into the multivariable regression model thus avoiding the exclusion of variables that may be marginally significant at selection but become statistically significant in the final adjusted analysis. Statistical analyses were performed using STATA version 14 (Stata Corp, College Station, TX).

## Results

From 2003 to 2012, we screened 1028 men, of whom 979 (95.2%) were eligible and all agreed to be interviewed. 976 participants (median: 28 years, range: 17–65 years) were included in the final analysis after excluding 3 questionnaires with many missing responses. All the men in the study reported vaginal intercourse and less than 4% reported oral or anal intercourse. Table 1 compares the socio-demographic characteristics and condom use behaviours in the time periods before and after brothel closure. Compared to the men before brothel closure, those in the post-closure period tended to be younger (65% versus 52% < 30 years), single (54% versus 44%), non-regular drinkers (90% versus 84%) and of higher income (72% versus 40% earning ≥$US 100 per month). Men in the post-brothel closure period were less likely to report consistent condom use for vaginal intercourse in the last 6 months (36% versus 63%, *p* < 0.001) and at last intercourse (25% versus 58%, *p* < 0.001) with a sex worker. The provision of condoms for last intercourse had shifted predominantly from the sex worker (91%) before closure to the men (65%) after brothel closure.

**Table 1:** Social-demographic and condom use characteristics of men who buy sex before and after brothel closure

Variables	Total *N* = 976		Brothel closure				*p*-value
			Before (*N* = 757)		After (*N* = 219)		
	n	%	n	%	n	%	
Sociodemographic characteristics							
Age (years)^a^							
< 20	31	(3.2)	20	(2.6)	11	(5.1)	0.003
20–29	504	(51.7)	373	(49.3)	131	(60.1)	
30–39	320	(32.8)	267	(35.3)	53	(24.3)	
≥ 40	120	(12.3)	97	(12.8)	23	(10.6)	
Median age (years), (range)	28 (17–65)		29 (17–65)		26 (17–57)		
Education level^a^							
No formal education	37	(3.8)	30	(4.0)	7	(3.2)	0.605
Primary (1–6 years)	303	(31.1)	240	(31.8)	63	(28.8)	
Secondary (7–12 years)	596	(61.1)	454	(60.1)	142	(64.8)	
College and university	39	(4.0)	32	(4.2)	7	(3.2)	
Marital status^a^							
Never married	452	(46.5)	335	(44.3)	117	(53.9)	< 0.001
Married	463	(47.6)	379	(50.1)	84	(38.7)	
Divorced/separated	32	(3.3)	30	(4.0)	2	(0.9)	
Widowed	26	(2.7)	12	(1.6)	14	(6.5)	
Income per month (USD)^a^							
< 100	503	(53.0)	444	(59.9)	59	(28.4)	< 0.001
100–200	302	(31.8)	201	(27.1)	101	(48.6)	
201–300	80	(8.4)	54	(7.3)	26	(12.5)	
> 300	64	(6.7)	42	(5.7)	22	(10.6)	
Alcohol consumption^a^							
No	52	(5.3)	52	(6.9)	0	(0.0)	< 0.001
Current occasional drinker	775	(79.5)	579	(76.6)	196	(89.5)	
Regular drinker	148	(15.2)	125	(16.5)	23	(10.5)	
Sex workers							
Types of sex workers^a^							
Brothel based only	579	(70.8)	470	(77.1)	109	(52.4)	< 0.001
Non-brothel based only	206	(25.2)	110	(18.0)	96	(46.2)	
Both brothel and non-brothel based	33	(4.0)	30	(4.9)	3	(1.4)	
Condom use with sex workers^b^							
Condom use for vaginal sex in the last 6 months							
Yes	553	(56.7)	474	(62.6)	79	(36.1)	< 0.001
No	423	(43.3)	283	(37.4)	140	(63.9)	
Condom use for vaginal sex in the last week^a^							
Yes	520	(63.0)	409	(66.5)	111	(52.9)	< 0.001
No	305	(37.0)	206	(33.5)	99	(47.1)	
Who provided condom for the last vaginal sex?^a^							
Sex workers^b^	381	(88.4)	375	(90.6)	6	(35.3)	< 0.001
Own self	50	(11.6)	39	(9.4)	11	(64.7)	

*USD*; United States dollar, *STD/HIV*; sexually transmitted diseases/human immunodeficiency virus

^a^Exclude missing values

^b^Include brothel and non-brothel based sex workers

**Fig. 1:**
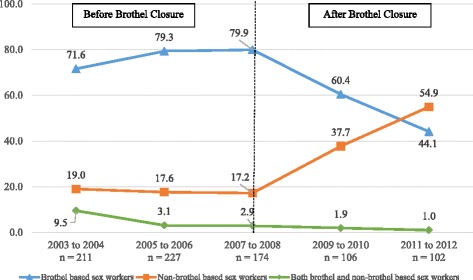
Percentage of men who patronised brothel and non-brothel-based sex workers in the last three months. Brothel based sex workers: Chi-square test for trend in proportions, *p* < 0.001. Non-brothel based sex workers: Chi-square test for trend in proportions, *p* < 0.001. Both brothel and non-brothel based sex workers: Chi-square test for trend in proportions, *p* < 0.001. Numbers in graph exclude missing values. Overall, less than 4% of all the men between 2003 and 2012 had sex with both brothel and non-brothel based sex workers

Figure 1 shows trends in men buying sex by type of sex worker over the 10-year period. In the 6-year period before brothel closure, self-reported sex with brothel-based sex workers only in the last 3 months hovered between 72% and 80%. After brothel closure it decreased by almost half to 44% (*p* < 0.001) in 2011–2012. In contrast, self-reported sex with non-brothel-based sex workers increased almost 3-fold over the same time period, ranging from 17% to 19% before brothel closure to 55% (*p* < 0.001) in 2011–2012. Overall, less than 4% of men bought sex from both brothel-based and non-brothel-based sex workers over the 10-year period.

**Fig. 2:**
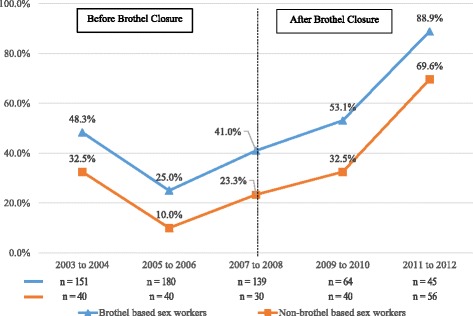
Prevalence of unprotected sex with brothel and non-brothel-based sex workers in the last six months. Brothel based sex workers: Chi-square test for trend in proportions, *p* < 0.001. Non-brothel based sex workers: Chi-square test for trend in proportions, *p* < 0.001. Numbers in graph exclude missing values

**Fig. 3:**
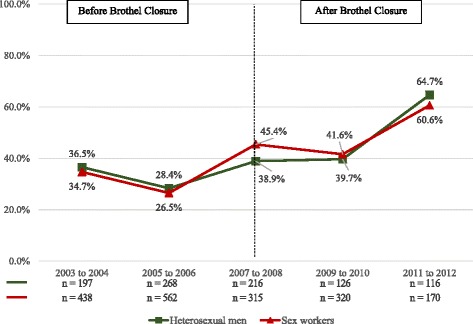
Comparison of prevalence of unprotected sex in the last week between sex workers and clients. Heterosexual men: Chi-square test for trend in proportions, *p* < 0.001. Sex workers: Chi-square test for trend in proportions, *p* < 0.001. Comparison between heterosexual men and sex workers engaging in unprotected sex in the last week at 3 time points; 2003 to 2004, 2007 to 2008 and 2011 to 2012: Chi-square, *p* = 0.118. Numbers in graph exclude missing values

**Fig. 4:**
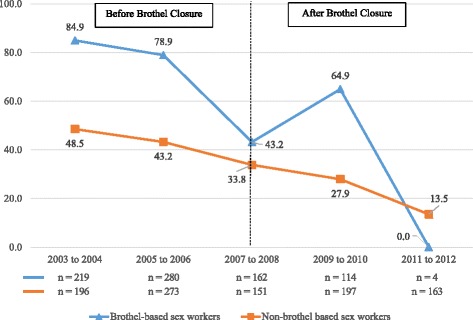
Percentage of brothel and non-brothel-based sex workers who refused unprotected sex with male clients who declined condom use. Brothel based sex workers: Chi-square test for trend in proportions, *p* < 0.001. Non-brothel based sex workers: Chi-square test for trend in proportions, *p* < 0.001. Numbers in graph exclude missing values

The percentage of men reporting unprotected intercourse with brothel-based workers in the last 6 months increased from 48% in 2003–2004 before brothel closure to 89% in 2011–2012 after brothel closure. This almost 2-fold increase was also found for men who reported unprotected intercourse with non-brothel-based sex workers, where it increased from 33% to 70% over the same time period (Fig. 2). The rising trend in unprotected intercourse in the last week with all sex workers by the men from 37% in 2003–2004 to 65% in 2011–2012 corresponded closely with the increase in self-reported unprotected intercourse (35% to 61%) by the sex workers (*n* = 1805) over the same period (Fig. 3). The percentage of sex workers who always refused unprotected intercourse when clients declined to use condoms decreased markedly from 67% in the first time period before brothel closure to 13% (*p* < 0.001) in the last period after brothel closure. The same decreasing trend was observed for both brothel and non-brothel-based sex workers. (Fig. 4).

**Fig. 5:**
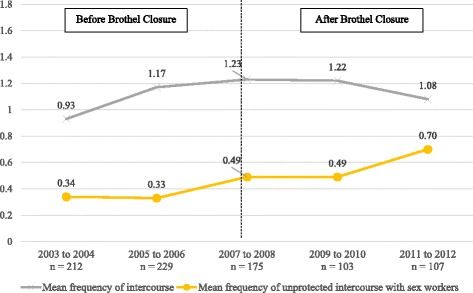
Mean frequency of intercourse and unprotected intercourse with sex workers in the last week. Mean frequency of intercourse: Modified Poisson regression with robust sandwich variance, *p* < 0.001. (Adjusted for age, marital status, income per month, alcohol consumption and self-perceived risk of getting STD/HIV from sex workers). Mean frequency of unprotected intercourse with sex workers: Modified Poisson regression with robust sandwich variance, *p* < 0.001. (Adjusted for age, marital status, income per month, alcohol consumption and self-perceived risk of getting STD/HIV from sex workers). Numbers in graph exclude missing values

The mean frequency of vaginal intercourse with sex workers in the last week reported by the men declined from a peak of 1.23 in 2007–2008 before brothel closure to 1.08 in 2011–2012 (mean difference: 0.15, *p* < 0.001). The mean frequency of unprotected vaginal intercourse increased more markedly from 0.34 in 2003–2004 to 0.70 times in 2011–2012 (mean difference: 0.36, *p* < 0.001), resulting in a narrowing gap between vaginal and unprotected vaginal intercourse over time (Fig. 5). During the first time period before brothel closure, unprotected intercourse constituted 37% of the total number of times of vaginal sexual encounters in the last week. This increased to 65% of the sexual encounters at the last time period after brothel closure. Comparing the pre- and post-closure periods, HIV prevalence in the heterosexual men showed a significant decline from 26% in 2003–2004 to 4.8% in 2007–2008 and 0 case in 2009–2010 before increasing to 5.6% in 2011–2012.

In the univariate analysis, significant associations were found between unprotected intercourse in the last 6 months and brothel closure, income, alcohol consumption and self-perceived risk of getting STIs/HIV from sex workers (Table 2). The strongest significant association with unprotected intercourse was found for brothel closure. After brothel closure, 63.9% of the men engaged in unprotected intercourse compared to 37.4% before brothel closure (PR: 1.71, 95% CI: 1.49–1.96). Adjustment for income, alcohol consumption and self-perceived risk did not materially alter the prevalence ratio (aPR: 1.65, 95% CI: 1.40–1.94). Compared with the pre-brothel closure period, men were 1.65 times as likely to report unprotected intercourse after brothel closure in the adjusted analysis.

**Table 2:** Brothel closure, social-demographics characteristics, risk perception, and practice of unprotected sex among men who buy sex

Variables	Unprotected sex with sex workers^c^ in the last 6 months				Crude PR	95% CI	Adjusted PR	95% CI
	Total (*N* = 976)	Yes (*N* = 423)		*p*-value				
	n	n	%					
Time period								
Closure of brothels								
Before closure (2003 to 2008)	757	283	(37.4)	< 0.001	1		1	
After closure (2009 to 2012)	219	140	(63.9)		1.71	1.49–1.96	1.65	1.40–1.94
Sociodemographic characteristics								
Age (years)^a^								
< 20	31	16	(51.6)	0.934^‡^	1		1	
20–29	504	216	(42.9)		0.83	0.58–1.18	0.96	0.62–1.49
30–39	320	136	(42.5)		0.82	0.57–1.19	1.05	0.66–1.67
≥ 40	120	54	(45.0)		0.87	0.59–1.29	1.11	0.69–1.81
Education level^a^								
No formal education	37	15	(40.5)	0.858^‡^	1		1	
Primary (1–6 years)	303	135	(44.6)		1.10	0.73–1.66	1.11	0.71–1.74
Secondary (7–12 years)	596	255	(42.8)		1.06	0.71–1.58	1.05	0.68–1.64
College and university	39	17	(43.6)		1.08	0.63–1.83	1.17	0.66–2.06
Marital status^a^								
Never married/single	452	200	(44.3)	0.607	1		1	
Married/ever married^b^	521	222	(42.6)		0.96	0.83–1.11	0.89	0.74–1.07
Income per month (USD)^a^								
≤ 200	805	360	(44.7)	0.004	1		1	
> 200	144	46	(31.9)		0.71	0.56–0.92	0.69	0.54–0.88
Alcohol consumption^a^								
No and occasional drinker	827	334	(40.4)	< 0.001	1		1	
Regular drinker	148	88	(59.5)		1.47	1.26–1.72	1.62	1.36–1.92
Risk perceptions								
Own chance of getting STD/HIV from sex workers^a,c^								
None to low	438	214	(48.9)	< 0.001	1		1	
Moderate to very high	517	190	(36.8)		0.75	0.65–0.87	0.83	0.71–0.98

*USD*; United States dollar, *STD/HIV*; sexually transmitted diseases/human immunodeficiency virus, *PR*; prevalence ratio, *CI*; confidence interval

^a^Exclude missing values

^b^Include divorced/separated/widowed

^c^Include brothel and non-brothel based sex workers

^‡^Chi-square test for trend in proportions

## Discussion

Behavioural surveillance of cross-sectional samples of male clients of female sex workers attending a public health centre for VCCT over a 10-year period—including 6 years before and 4 years after brothel closure—found that brothel closure was independently associated with a 1.7-fold increase in unprotected intercourse with sex workers during the last 6 months. Buying sex from brothel-based sex workers by these men decreased by almost half to 44% after brothel closure but buying sex from non-brothel-based sex workers increased markedly by almost 3-fold to 55%.

Despite the ban on brothels with criminalisation and suppression of direct sex work, our finding of a significant proportion (44%) of men still reporting sex with brothel-based sex workers suggests that the latter were working illegally/underground. This corroborates with other qualitative studies in Cambodia [21, 23, 24] which found brothel owners to be operating illegally from other non-brothel based venues. The marked rise in unprotected intercourse following brothel closure might be explained by the lack of access to condoms [23]. This is supported by our finding in which only 35% of the sex workers provided condoms after brothel closure. In contrast, before brothel closure, almost all sex workers (91%) provided condoms at the most recent protected intercourse, suggesting their ready access to free condoms from the government’s 100% condom program, alongside non-governmental organisations’ health promotion programs. The Law on the Suppression of Human Trafficking that resulted in the ban on brothels and criminalisation of sex work rendered infeasible the implementation of the 100% condom use program in the following ways. First, the brothel ban led to the displacement of brothel-based sex workers to illegal hidden venues such as entertainment establishments and the streets that hampered outreach HIV prevention efforts such as condom distribution to the sex workers. Second, the criminalisation of sex work led to more harassment and arrests of the sex workers. Hence, many sex workers did not carry condoms out of fear that condoms would be used as evidence of sex work [21]. Similarly, entertainment venues did not provide condoms in case they provided evidence of sex work in their premises.

The decline in condom use was consistent with findings from another study on female entertainment workers in Cambodia which found lower condom use in 2014 than in 2012 [32]. Other findings in our study on the increase in sex with non-brothel-based workers by the men following the brothel ban corroborated with Page et al.’s [22] findings of sex work shifting to entertainment establishments.

### Limitations and strengths

Our study has some limitations. Our study in a clinic setting—heterosexual men attending the health centre for VCCT—limits the generalisability of the findings to the general population of heterosexual men who engaged in transactional sex. However, our primary aim was not to extrapolate our findings, but to assess the impact of brothel closure on high-risk heterosexual men attending the only government Health Centre with VCCT service in Siem Reap. We believe our findings can be generalised to men attending Mondol Moi Health Centre because we took steps to ensure that the men were randomly selected. To assess the representativeness of our sample, we took a systematic random sample of 2481 patient records from the retrospective data set of all VCCT attendees between 2003 and 2009. The men in this data set did not differ significantly in age (*p* > 0.55) and marital status (*p* > 0.07) from the samples in our study over the same time period. Attendance at the centre could be affected by increasing public awareness on VCCT. However, the program or VCCT criteria did not change during the study period. Self-reported behaviour could be biased by social desirability and recall. But, the high percentage of men reporting unprotected intercourse with both brothel and non-brothel-based sex workers suggests that social desirability bias, if any, was minimal. Moreover, steps were taken to reduce this bias. To reduce errors in interpretation from recall bias, we collected and triangulated data from different time periods—“last sex” activity, “sex in last week”, and “last 6 months”. As our results were based on cross-sectional surveys, the associations between brothel closure and sexual risk behavioural outcomes do not imply causality. However, repeat measurements of the same variables in serial random cross-sectional samples over a 10-year period allows the comparison of trends before and after brothel closure.

This study, to our knowledge, is the first comprehensive analysis of trends in sexual risk behaviours of male clients of female sex workers following the ban on trafficking and brothel-based sex work in Cambodia. To date, all published studies on the impact of criminalisation of sex work and brothel ban have focused on female sex workers. While it is important to study sex workers, research on them in settings where sex work is criminalised might be hindered by difficulty in access and in getting them to participate in research for fear of arrest. The National AIDS Authority itself highlighted the difficulty distinguishing those who mainly sell sex from those doing so occasionally in conducting HIV-related behavioural surveillance [33]. Despite legislation, men buying or soliciting sex are rarely criminalised in Cambodia for sexual acts with persons over the age of 18. They often confidentially declare their engagement in transactional sex even from illegal brothels, particularly when they are attending a clinic for VCCT. In fact, a significant proportion (52%) of the men in our study reported having sex with brothel-based sex workers even after the law had been implemented to ban brothels. Another study strength is the 10-year period of data collection in the same setting by one interviewer (ST, 2005–2012) and another (MC, 2002–2004) before and after implementation of the brothel ban. Other strengths include assessing the frequency of unprotected vaginal intercourse itself rather than just determining the percentage of men not using condoms. We also corroborated sexual behavioural data of the men with that of sex workers collected over the same time period. The availability of laboratory-confirmed HIV for men over the 10-year period is a methodological strength. However, given our small yearly samples of men coupled with the finding of an increase in HIV prevalence at only one-time period after brothel closure, we cannot conclude that brothel closure is associated with an increase in HIV prevalence. Future research should monitor HIV in larger samples of male clients of sex workers.

## Conclusions

Our findings are consistent with a growing body of evidence that forced brothel closure coupled with criminalisation of sex work is associated with an increase in unprotected intercourse that may facilitate HIV/STI transmission [9, 25]. Contrarily, studies on decriminalisation of sex work [34, 35] coupled with delivery of culturally appropriate STI prevention education and referral services to Asian immigrant massage parlour workers in the US [34] and Asian female brothel-based sex workers in Australia [35] have found an increase in condom use and a decrease in STI incidence [36]. A recent modelling study also found that decriminalisation of sex work could avert 33% to 46% of HIV infections worldwide [37]. Given our study findings, coupled with positive outcomes from other research on decriminalisation of sex work, and a lack of evidence for the effectiveness of criminalisation of sex work in reducing sex trafficking [38, 39], the part of the anti-trafficking law banning brothels in Cambodia should be reviewed. A policy that provides a health-promoting work environment for sex workers to practise safe sex with their clients is critical. Cambodia has succeeded in promoting condom use and reducing HIV among brothel-based sex workers after the implementation of the 100% condom use policy in brothels in 2001. It is crucial for this success to be sustained.

## Abbreviations

AIDS: Acquired immune deficiency syndrome
aPR: Adjusted prevalence ratio
BSS: National behavioural sentinel surveillance
EIA: Enzyme immunoassay
HIV: Human immunodeficiency virus
IDRC: International Development Research Centre
NGO: Non-governmental organization
PR: Prevalence ratio
STD: Sexually transmitted diseases
STI: Sexually transmitted infections
VCCT: Voluntary confidential counselling and testing
